# Solid and macroporous Fe_3_C/N-C nanofibers with enhanced electromagnetic wave absorbability

**DOI:** 10.1038/s41598-018-35078-z

**Published:** 2018-11-15

**Authors:** Huihui Liu, Yajing Li, Mengwei Yuan, Genban Sun, Qingliang Liao, Yue Zhang

**Affiliations:** 1 Beijing Key Laboratory of Energy Conversion and Storage Materials and College of Chemistry, Beijing Normal University, Beijing, 100875 China; 2 State Key Laboratory for Advanced Metals and Materials, School of Materials Science and Engineering, University of Science and Technology Beijing, Beijing, 100083 China

## Abstract

A series of solid and macroporous N-doped carbon nanofibers composed of Fe_3_C nanoparticles (named as solid Fe_3_C/N-C NFs, solid Fe_3_C/N-C NFs-1, solid Fe_3_C/N-C NFs-2, macroporous Fe_3_C/N-C NFs, macroporous Fe_3_C/N-C NFs-1 and macroporous Fe_3_C/N-C NFs-2, respectively) were prepared through carbonization of as-electrospun nanofiber precursors. The results show that the magnetic Fe_3_C nanoparticles (NPs) dispersed homogeneously on the N-doped carbon fibers; as-prepared six materials exhibit excellent microwave absorption with a lower filler content in comparison with other magnetic carbon hybrid nanocomposites in related literatures. Particularly, the solid Fe_3_C/N-C NFs have an optimal reflection loss value (*RL*) of −33.4 dB at 7.6 GHz. For solid Fe_3_C/N-C NFs-2, the effective absorption bandwidth (EAB) at *RL* value below −10 dB can be up to 6.2 GHz at 2 mm. The macroporous Fe_3_C/N-C NFs have a broadband absorption area of 4.8 GHz at 3 mm. The EAB can be obtained in the 3.6–18.0 GHz frequency for the thickness of absorber layer between 2 and 6 mm. These Fe_3_C–based nanocomposites can be promising as lightweight, effective and low-metal content microwave absorption materials in 1–18 GHz.

## Introduction

Following rapid development of electronic science and technology, widespread use of electronic devices in wireless communications, high-frequency circuit components and other related fields^1–3^. Considerable efforts have been put to prepare the effective electromagnetic (EM) samples due to its application in the military stealth technology^4–6^. In recent years, with the increasing attention on the prevention and control of electromagnetic pollution and the increasing requirements of military weapons, microwave absorbing samples with a low thickness, light density, high reflection loss at a wide frequency range and corresponding stealth technology are urgently needed^7–9^. And a number of studies have endeavored to the development of such materials^10,11^. For example, the flower-like CuS hollow materials were successfully prepared by a simple solvothermal procedure. This material can reach a lowest *RL* value of −31.5 dB with 30 wt% filler loading^3^. The Ni−CNT nanocomposites obtained the lowest *RL* values (−30.0 dB) at 2.0 mm thickness^10^. The CNFs/Fe absorbers exhibited super-duper EM wave absorbing ability on the basis of a lowest *RL* value (−67.5 dB) with merely 5 wt% filler loading^11^. Among these microwave-absorbing materials, carbon-based composite microwave-absorbing materials comprising of carbon and magnetic particles have been attracting increasing attention, including Fe_3_O_4_ multi-walled carbon nanotube (CNTs)^9^, Fe_3_C/CNT nanocomposites^12^ and porous Co/CNTs^13^. It is worth mentioning that our group prepared the cobalt/N-doped carbon nanocomposites with two morphologies using electrospinning and annealing methods^14^. The solid Co/N-doped carbon nanocomposites show a strong absorbing property of −24.5 dB at 3 mm with 10 wt% filler loadings. Because these materials not only have large EM losses, which can effectively improve EM wave absorption, but also can overcome the disadvantages of magnetic components with a high density^13–17^. The Fe_3_C nanoparticles possess satisfactory stability and outstanding magnetic properties^18–20^. Some studies have shown that the combination of Fe_3_C and carbon nanofibers can significantly improve the microwave absorption performance^21^. In order to meet the requirements of absorbing materials including light density, thin thickness, and high reflection loss at a wide frequency range, we have prepared the macroporous Fe_3_C/N-doped carbon nanofibers with a novel macroporous structure. The experimental results suggested that the macroporous Fe_3_C/N-doped carbon nanofibers were endowed with a lighter weight and a broader bandwidth contrasted to the solid Fe_3_C/N-doped carbon nanofibers. And the effective microwave absorption of macroporous Fe_3_C/N-doped carbon nanocomposites can be obtained in the 3.6–18.0 GHz frequency range. At the same time, we also prepared the solid Fe_3_C/N-doped carbon nanocomposites, which exhibit superior microwave absorbability with a small *RL* value of −34.9 dB. The Fe_3_C/N-doped carbon composite nanofibers achieve a significant advancement in EM wave absorbing ability at the same 10 wt% filler content compared with the Co/N-doped carbon composite nanofibers^14^. This may be caused by the unique magnetic properties of Fe_3_C nanoparticles and the better impedance matching effect between Fe_3_C and carbon nanofibers^13,18^. In recent years, the electrospinning technology has become one of the popular techniques in fabricating nanofibers, because it is a simple and mature technology that can be applied on a large scale^22,23^. For instance, carbon fibers consist of Fe_3_O_4_ particles were prepared by electrospinning techniques. The sample reached the minimum *RL* value of −44.0 dB^24^. Park *et al*. synthesized Ni–Fe composite carbon fibers by electrospinning process and the materials showed good electromagnetic wave absorbability^25^.

In this work, a series of solid and macroporous Fe_3_C/N-doped carbon hybrid nanocomposites have been obtained by carbonizing as-spun polyacrylonitrile (PAN)-based nanofiber precursors, which were synthesized by spinning a PAN/DMF (N, N-dimethyl formamide) solution containing iron acetylacetonate. The six nanocomposites with 10 wt% filler loading showed an exceedingly good microwave absorption property. The as-prepared lightweight magnetic carbon hybrid nanocomposites will be valuable in different EM absorbing areas due to their superior EM absorption performance.

## Results and Discussion

**Figure 1:**
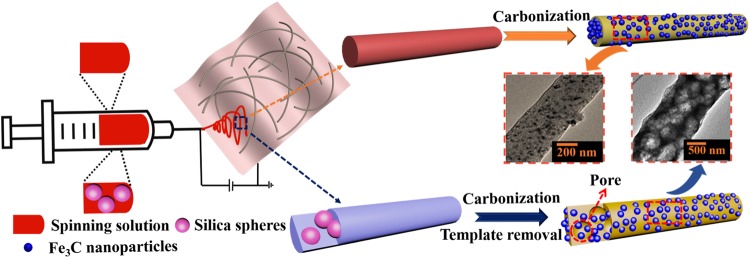
Schematic illustration of the synthesis of the two Fe_3_C/N-doped carbon nanocomposites.

**Figure 2:**
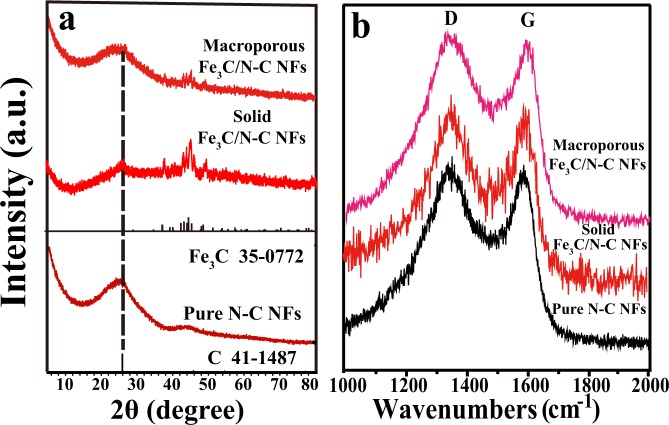
(**a**) XRD patterns and (**b**) Raman spectra of the as-obtained two representative Fe_3_C/N-C nanofibers and pure N-C nanofibers.

Figure 1 illustrates the preparation process of solid and macroporous Fe_3_C/N-doped carbon nanofibers by using electrospinning and calcination treatment. As shown in Fig. 1, the precursor nanofibers were fabricated using electrospinning method^11^. According to the literature^21,26,27^, in the heating process of the tube furnace, the polyacrylonitrile in the precursor fiber decomposes at 250–300 °C. The main decomposition products are hydrogen cyanide, ammonia and carbon, etc., however, the fiber morphology remains. Iron acetylacetonate starts to decompose into iron oxide at 280 °C. These Fe_3_C particles are distributed on the surface and inside of the nanofiber. As the temperature increases, the iron oxide reacts with carbon to form Fe_3_C nanoparticles, then the final sample of N-doped carbon nanofibers composed of Fe_3_C nanoparticles was produced^28,29^. The crystallographic features of the synthesized representative products were examined by XRD and Raman spectrometer (Fig. 2). In Fig. 2a, the peak at 2θ = 26.5° match with XRD patterns of the standard graphite carbon (JCPDS No. 41–1487)^30,31^. Other obvious diffraction peaks at 37.8°, 39.9°, 40.8°, 43.0°, 43.8°, 44.7°, 45.1°, 46.0°, 48.7° and 49.3° can be indexed to the (021), (200), (120), (121), (210), (022), (103), (211), (113) and (122) planes of Fe_3_C phase (JCPDS No. 75–0910), respectively, revealing that the Fe_3_C nanoparticles and nanocrystalline graphite are successfully synthesized by the carbonization method^32,33^. In the Raman spectra depicted in Fig. 2, two broad peaks at 1342 cm^−1^ and 1580 cm^−1^ correspond to the D band and G band from carbon. As is known to all, the ratios of I_D_/I_G_ indicate the carbon disorder degree^34,35^. In Fig. 2b, we calculated the value of I_D_/I_G_ to be 1.08:1 for solid Fe_3_C/N-doped carbon nanocomposites, 1.05:1 for macroporous Fe_3_C/N-doped carbon nanocomposites and 1.03:1 for pure N-doped carbon nanofibers, respectively. It implies that the disorder degree of the carbon in our materials has greatly increased compared with pure N-doped carbon nanofibers. Moreover, the carbon with plenty of defects can be considered as effective polarization center under electromagnetic field^36^.

**Figure 3:**
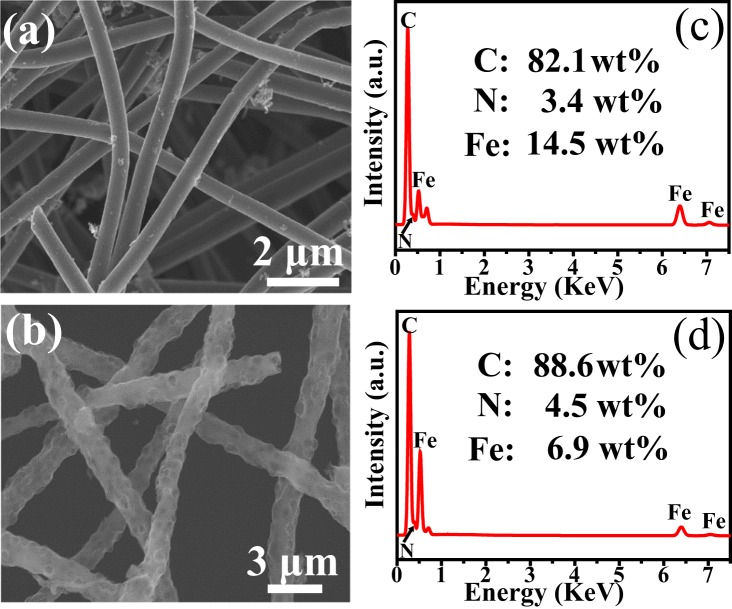
(**a**,**b**) SEM images, (**c**,**d**) EDS spectra of the representative Fe_3_C/N-doped carbon nanocomposites with two structures, respectively.

The SEM images (Fig. S1a,b) show the typical morphology of the as-obtained materials. The lengths of these two representative nanofibers could beyond several micrometers. The average diameters of solid Fe_3_C/N-C nanofibers are 500 nm, whereas the average diameters of macroporous Fe_3_C/N-C nanofibers are 1 μm (Fig. 3a,b). Their component contents are measured by EDS analysis (Fig. 3c,d). The results reveal the occurrence of C, N and Fe elements in as-synthesized two composites. For solid Fe_3_C/N-doped carbon nanofibers, the respective C, N and Fe mass fractions are 82.1%, 3.4% and 14.5%, while in the macroporous Fe_3_C/N-doped carbon nanofibers, the respective C, N and Fe mass fractions are 88.6%, 4.5% and 6.9%. The EDX mapping images and EDS spectra of solid Fe_3_C/N-doped carbon NFs-1, solid Fe_3_C/N-doped carbon NFs-2, macroporous Fe_3_C/N-doped carbon NFs-1 and macroporous Fe_3_C/N-doped carbon NFs-2 are shown in Fig. S2. For solid Fe_3_C/N-C NFs-1, the respective C, N and Fe mass fractions are 95.7%, 0.5% and 3.8%. As for solid Fe_3_C/N-C NFs-2, the respective C, N and Fe mass fractions are 80.6%, 4.4% and 15.0%. For macroporous Fe_3_C/N-C NFs-1, the respective C, N and Fe mass fractions are 90.0%, 4.8% and 5.2%. For macroporous Fe_3_C/N-C NFs-2, the respective C, N and Fe mass fractions are 86.6%, 5.0% and 8.4%. For the as-prepared solid and macroporous Fe_3_C/N-doped carbon nanofibers, as the amount of iron acetylacetonate of the spinning solution increases, the iron content of the final products also increases.

**Figure 4:**
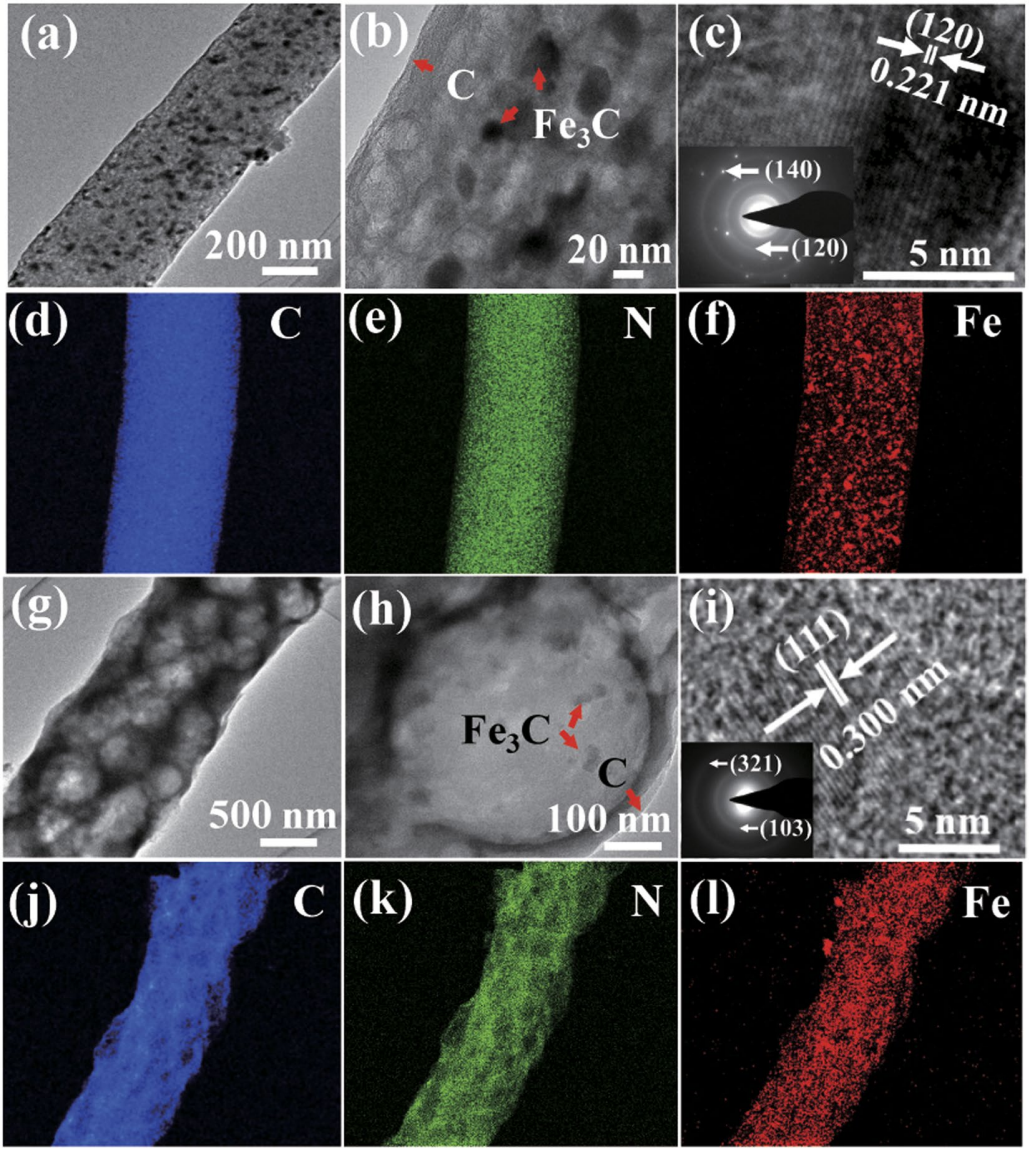
Morphologies and elemental mapping images of the representative Fe_3_C/N-doped carbon nanocomposites with two structures: (**a**,**b**,**g**,**h**) TEM images, (**c**,**i**) HRTEM images, the insets of (**c**,**i**) corresponding SAED pattern, (**d**–**f**,**j**–**l**) EDX mapping images.

**Figure 5:**
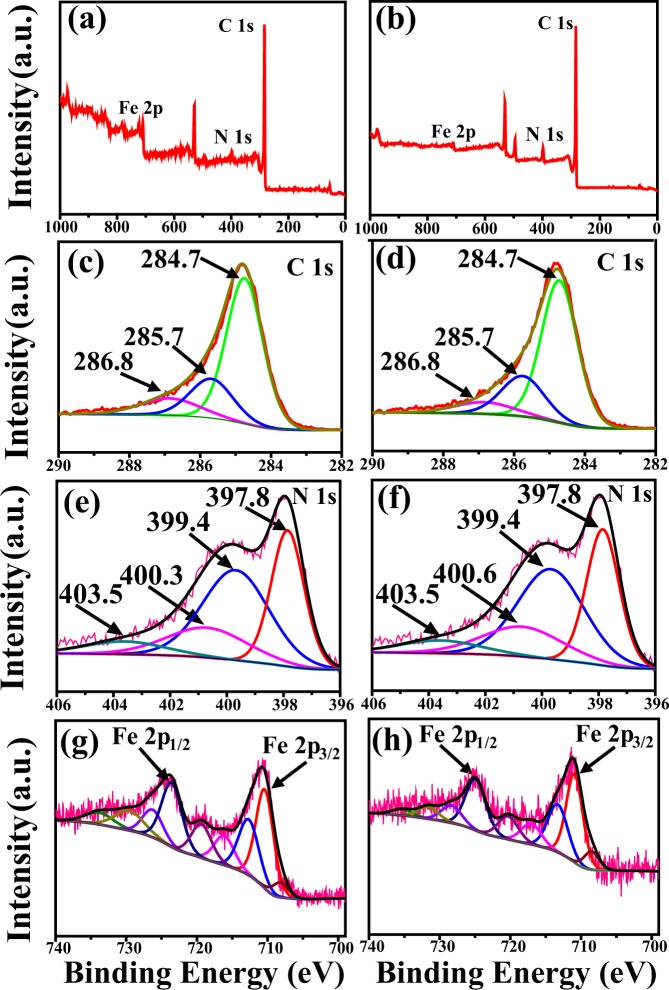
XPS spectra of (**a**,**b**) Survey scans, (**c**,**d**) C 1s spectrum, (**e**,**f**) N 1s spectrum, (**g**,**h**) Fe2p spectrum of the representative Fe_3_C/N-doped carbon nanocomposites with two structures, respectively.

Figure 4 shows typical TEM photographs and EDX mapping images. It is obvious that numerous Fe_3_C NPs are uniformly distributed in the two prepared materials (Fig. 4a,g). Further, the particle sizes of the Fe_3_C NPs ranged from 20 to 50 nm (Fig. 4b,h). Moreover, the Fe_3_C NPs in the solid Fe_3_C/N-doped carbon nanofibers present a lattice spacing of 0.221 nm between adjacent lattice, corresponding to the (120) lattice plane (Fig. 4c), indicating a high crystallinity. The elemental mappings (Fig. 4d–f) reveal the uniform distribution of Fe_3_C nanoparticles throughout the nanofibers. Furthermore, the existence of C, N and Fe in the structure is further demonstrated by the peaks of N 1s, C 1s, and Fe 2p observed in Fig. 5. The HRTEM images in Fig. 4g,h, the TEM and STEM images in Fig. S1c,d demonstrate that the macroporous Fe_3_C/N-doped carbon nanofibers have a pore size of around 500 nm. In Fig. 4i, the lattice distance is 0.300 nm, corresponding to the (111) diffraction peak of Fe_3_C^37^. The inset of Fig. 4i displays a selected area electron diffraction (SAED) pattern and the clear diffraction rings represent the (321) and (103) planes of the Fe_3_C nanoparticles. Moreover, the EDX mapping results confirm that Fe_3_C NPs are decorated in the macroporous Fe_3_C/N-doped carbon nanofibers (Fig. 4j–l).

Figure 5 shows the survey spectra of the two representative nanocomposites. In the survey XPS spectra (Fig. 5a,b), the XPS peaks for C 1s, N 1s, and Fe 2p indicate the existence of these elements in these synthesized nanomaterials. Figure 5c,d represent the XPS spectra of C 1s for as-fabricated nanofibers. The C 1s spectrum includes three peaks that can be indexed to C-C at around 285.7 eV, C-N at around 284.7 eV and C-O at around 286.8 eV, respectively^38,39^. Figure 5e,f display N 1s spectra of the as-fabricated products. The N 1s spectrum include four different nitrogen atoms: pyridinic N (397.8 eV), pyrrolic N (399.4 eV), quaternary N (400.3 and 400.6 eV), and oxidized N (403.5 eV)^33^. The peaks at 708.5 eV is due to Fe_3_C. The other peaks shown in the spectra of Fe 2p in Fig. 5g could be divided into two pairs of peaks at around 710.4 and 724.4 eV, which represent Fe 2p_3/2_ and Fe 2p_1/2_, respectively^40,41^. This confirms the existences of Fe_3_C in as obtained materials. As shown in Fig. 5h, in the macroporous Fe_3_C/N-C NFs, the peaks at 708.5 eV correspond to Fe_3_C, and the other two major peaks at around 710.6 and 725.0 eV represent the Fe 2p_3/2_ and Fe 2p_1/2_, respectively.

**Figure 6:**
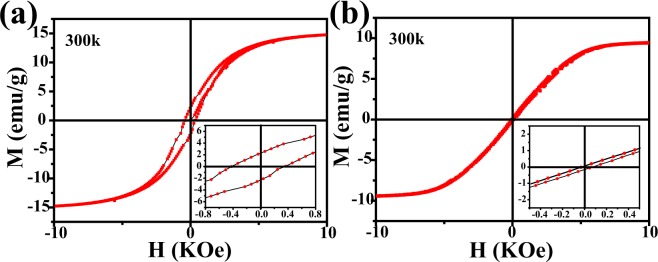
Hysteresis loops of (**a**) solid Fe_3_C/N-doped carbon nanocomposites and (**b**) macroporous Fe_3_C/N-doped carbon nanocomposites at 300 K.

Figure 6 illustrates the magnetic hysteresis loops of as synthetized Fe_3_C/N-C nanofibers materials measured at room temperature. From Fig. 6, it is not difficult to find that the two samples exhibit typical ferromagnetic properties at 300 K^42,43^. The saturation magnetization of the solid Fe_3_C/N-doped carbon nanocomposites reaches 14.8 emu/g and 9.4 emu/g for macroporous Fe_3_C/N-doped carbon nanocomposites. This phenomenon can be related to the higher iron contents for solid Fe_3_C/N-doped carbon nanocomposites^44^. As shown in Fig. 3c,d, the Fe mass fraction of the solid Fe_3_C/N-doped carbon hybrid nanocomposites (14.5%) is larger than that in macroporous Fe_3_C/N-doped carbon hybrid nanocomposites (6.9%). The specific surface area (SSA) of pure N-doped carbon nanofibers, solid and macroporous Fe_3_C/N-doped carbon nanofibers are 28.1, 144.3 and 30.6 m^2^ g^−1^, respectively (Fig. S3). As shown in Fig. S3, the two samples display IV N_2_ adsorption isothermals, indicating that mesoporous structures in these materials. The SSA of the obtained materials was computed by Brunauer-Emmett-Teller method. Due to the limitations of the method, the 500 nm macropores is too large to be represented in the specific surface area data, so the macropores has no remarkable impact on the SSA of the macroporous Fe_3_C/N-doped carbon nanocomposites. The Fe_3_C/N-doped carbon hybrid nanocomposites have a larger SSA compared with the pure N-doped nanofibers, which indicates that the mass fraction of iron plays a major role in the specific surface area of these materials^45,46^. Besides, the iron mass fraction of this macropores nanofibers is smaller than the nanofibers with solid structure^47,48^, so the SSA of the macroporous nanocomposites is smaller than the solid nanocomposites. As is known to all, coercivity force is considered as a momentous parameter to evaluating the magnetic performance. The EM wave absorbers possess a high coercivity force value might result in a resonance effect^49^. In addition, the coercivity force value is closely bound up with the metal content. As a result, a high metal content may cause a high coercivity^50^. Herein, the solid nanofibers with the higher iron content are endowed with the higher coercive force of 421 Oe, whereas the coercivity force of macroporous nanofibers are 46 Oe.

**Figure 7:**
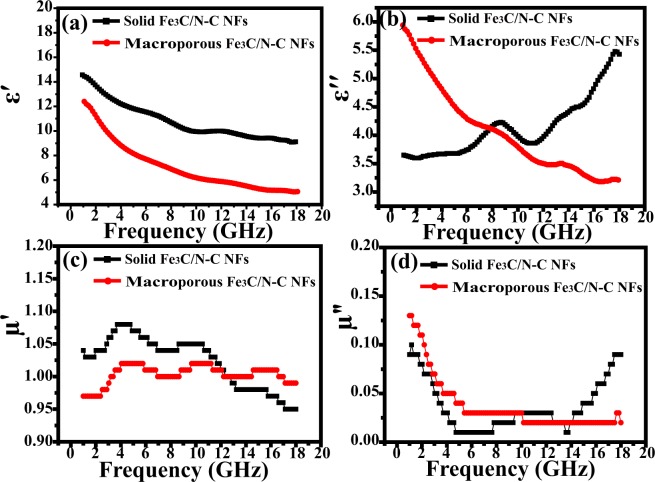
(**a**) ε′, (**b**) ε*″*, (**c**) μ′ and (**d**) *μ″* of the two representative Fe_3_C/N-doped carbon nanofibers with 10 wt% filler content.

Figure 7a,b denote the curve of the real and imaginary parts (ε′ and *ε″*) of the complex permittivity as a function of frequency. For the two Fe_3_C/N-doped carbon hybrid nanocomposites, the *ε*′ values decrease from 14.6 to 9.12 and 12.6 to 5.2 in 1–18 GHz, respectively. The *ε″* value of macroporous Fe_3_C/N-C NFs decreases from 6.0 to 3.2, while the *ε″* values of solid Fe_3_C/N-C NFs increases from 3.65 to 5.43 and exhibits a peak in the 6.0–11.0 GHz ranges, which reflects a relatively high dielectric loss^51^. In contrast to the macroporous nanocomposites, the solid nanocomposites have a larger *ε*′ value, suggesting that higher appropriate conductivity is obtained. As shown in Fig. 7c,d, the real (*μ*′) and imaginary (*μ″*) part of the complex permeability for two samples remains constant, so we suppose that the complex permittivity may play an important role in the EM absorbing ability of the two nanofibers.

**Figure 8:**
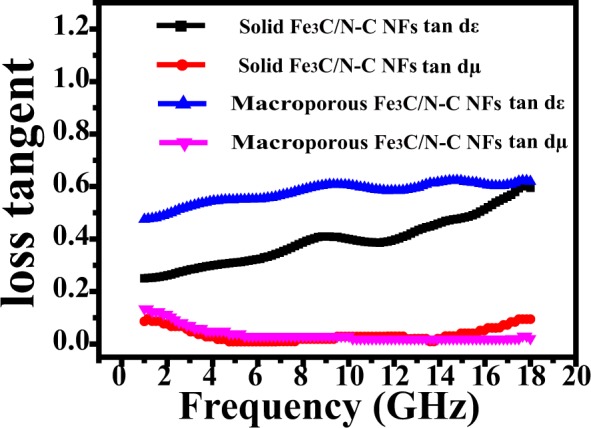
*tan d*_*ε*_ and *tan d*_*μ*_ for the two representative Fe_3_C/N-doped carbon nanofibers with 10 wt% doping content.

**Figure 9:**
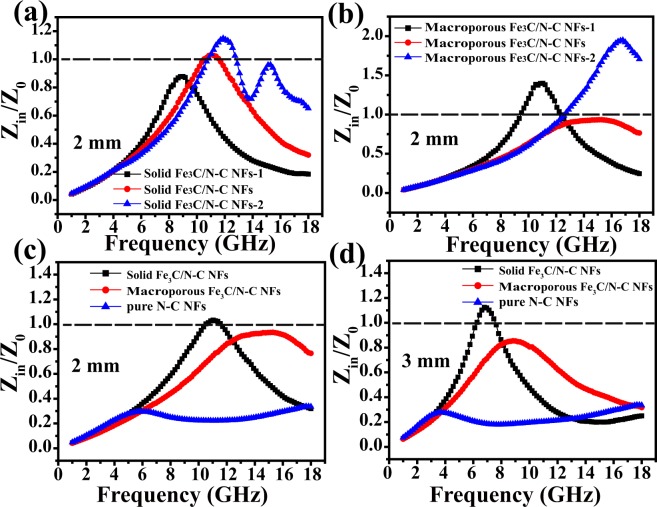
Calculated modulus of normalized characteristic impedance of (**a**) three as-obtained solid Fe_3_C/N-doped carbon nanocomposites at 2 mm (**b**) three as-synthesized macroporous Fe_3_C/N-doped carbon nanocomposites at 2 mm (**c**) the representative two Fe_3_C/N-C nanocomposites and pure N-C nanofibers at 2 mm (**d**) the representative two Fe_3_C/N-C nanocomposites and pure N-C nanofibers at 3 mm.

In order to investigate the main determinant for the as fabricate solid and macroporous nanofibers, we measured the dielectric dissipation factors *tan d*_*ε*_ = *ε″/ε*′ and magnetic loss tangent *tan d*_*μ*_ = *μ″/μ*′ of the two nanocomposites at 2 mm, respectively (Fig. 8). For the solid Fe_3_C/N-doped carbon nanofibers, the *tan d*_*ε*_ and *tan d*_*μ*_ curves have similar trends with the *ε″* and *μ″* curves (Fig. 7b,d), suggesting both dielectric loss and magnetic loss have an effect on the EM wave absorbing performance of the solid nanofibers, or, impedance matching^51,52^. Nevertheless, we didn’t see similar results for macroporous Fe_3_C/N-doped carbon nanofibers. On the other hand, we also calculated Z = |Z_in_/Z_0_| of as-prepared samples as shown in Fig. 9. Figure 9a displays the normalized characteristic impedance of three as-prepared solid Fe_3_C/N-doped carbon nanofibers at 2.0 mm. The experimental results show that the Z values of the solid Fe_3_C/N-C NFs and solid Fe_3_C/N-C NFs-2 are approximately 1.0 (The nearer to 1.0 for Z, the better the impedance matching properties), suggesting the rather good impedance matching properties^53^. Figure 9b displays the impedance matching performance of three as-prepared macroporous Fe_3_C/N-doped carbon nanofibers at 2.0 mm. It is obvious that the macroporous Fe_3_C/N-C NFs have more appropriate impedance matching properties compared with the other two macroporous Fe_3_C/N-doped carbon nanocomposites. Besides, we also compare the impedance matching performance of the two representative Fe_3_C/N-C nanofibers and pure N-C nanofibers with the thickness of 2 mm and 3 mm (Fig. 9c,d). The Z values of solid Fe_3_C/N-C NFs are close to 1, which represents a remarkable impedance matching performance. Especially, for solid Fe_3_C/N-C nanofibers, the appropriate components, synergistic effect of conductive N-doped carbon nanofibers and higher composition of magnetic Fe_3_C nanoparticles are propitious to their outstanding impedance matching properties. The Z values of macroporous Fe_3_C/N-C NFs are far away from 1.0, indicating a relatively worse impedance matching properties. The Z values of pure N-C nanofibers are near to 0, revealing nearly none impedance matching properties. Thus we speculate that the solid Fe_3_C/N-C NFs have better impedance matching properties which can lead leading to the excellent microwave-wave absorbing ability.

**Figure 10:**
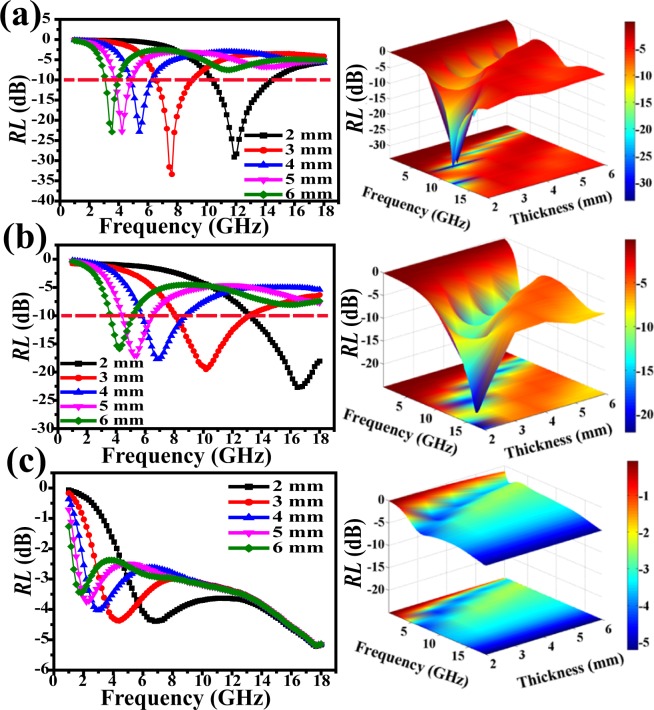
Simulated curves of EM wave reflection loss of (**a**) solid Fe_3_C/N-doped carbon nanocomposites (**b**) macroporous Fe_3_C/N-doped carbon nanocomposites, (**c**) pure N-doped carbon nanofibers with 10 wt% filler content.

The *RL* values were evaluated via measuring relative complex permittivity (*ε*_*r*_ = *ε*′ − *jε″*) and permeability (*μ*_*r*_ = *μ*′ − *jμ″*) based off of equations (1) and (2). The EM absorption property of solid Fe_3_C/N-doped carbon nanofibers with 10 wt% filler loading is shown in Fig. 10a. The respective optimal microwave absorption is achieved at 11.9 GHz for −29.1 dB (2 mm), 7.6 GHz for −33.4 dB (3 mm) and 5.4 GHz for −22.8 dB (4 mm), respectively. The EAB is 4.1 GHz (10.4–14.5 GHz) at 2.0 mm. In Fig. 10b, the macroporous Fe_3_C/N-doped carbon nanofibers with 10 wt% nanofiber loading shows an optimal *RL* value of −22.1 dB. The microwave absorbing bandwidths with *RL* lower than −10 dB are 4.8 GHz (8.2–13.0 GHz) at 3.0 mm. Apparently, the solid Fe_3_C/N-doped carbon nanocomposites present the optimal reflection loss value. Perhaps it is because the solid Fe_3_C/N-doped carbon nanofibers have the larger coercivity force and higher iron content^54,55^. Moreover, the proper impedance matching also plays a major role in its superior EM wave absorbing properties. As shown in Fig. 10c, the lowest *RL* value of the pure N-doped carbon nanofibers with 10 wt% doping amount is unable to achieve −10 dB at 2.0–6.0 mm. The EM absorbing ability of other two solid Fe_3_C/N-doped carbon nanofibers and two macroporous Fe_3_C/N-doped carbon nanofibers are shown in Fig. S4. In Fig. S4a, the optimal frequency value of solid Fe_3_C/N-C NFs-1 can reach −34.4 dB at 3.0 mm. It is worth mentioning that solid Fe_3_C/N-C NFs-2 with 6 mm possess an optimal *RL*_min_ of −34.9 dB. The effective absorption bandwidth can reach 6.2 GHz across 11.8–18.0 GHz (Fig. S4c). The macroporous Fe_3_C/N-C NFs-1 displays the optimal EM absorption of −32.8 dB among the three macroporous Fe_3_C/N-doped carbon nanocomposites (Fig. S4b). The macroporous Fe_3_C/N-C NFs-2 displays the strongest *RL* value of −15.0 dB (Fig. S4d). Table 1 concludes the EM-wave absorbing ability of these Fe_3_C/N-doped carbon composite nanofibers. Table 2 shows that the present solid Fe_3_C/N-C NFs-2 and macroporous Fe_3_C/N-C NFs exhibits the outstanding EM wave absorbing ability among the related Fe_3_C-based materials with the smallest fiber loadings. For example, Fe-Fe_3_C/C microspheres with 25 wt% doping amount gained an optimal *RL* of −18.8 dB^12^. The Fe_3_C/graphitic carbon with 70 wt% doping amount loading achieved the optimal *RL* value (−26 dB) at 2.0 mm^56^. The Fe_3_C/C nanocapsules with 40 wt% doping amount obtained the strongest *RL* of −38 dB and the EAB was only 2.0 GHz^57^. It is exhilarating to observe that these Fe_3_C/N-doped carbon nanocomposites possess an enhanced EM wave absorbing performance with the smallest fiber loadings^58^.

**Table 1:** Microwave absorbing ability of the as-prepared materials in 1–18 GHz.

Samples	Optimal *RL* Values (dB)	Matching Thickness (mm)	*RL* Values at 2 mm (dB)	Effective Bandwidth at 2 mm (GHz)
Solid Fe_3_C/N-C NFs	−33.4	3.0	−29.2	4.1
Solid Fe_3_C/N-C NFs-1	−34.4	3.0	−19.4	2.4
Solid Fe_3_C/N-C NFs-2	−34.9	6.0	−31.3	6.2
Macroporous Fe_3_C/N-C NFs	−22.1	2.0	−22.1	4.6
Macroporous Fe_3_C/N-C NFs-1	−32.8	5.0	−17.2	2.5
Macroporous Fe_3_C/N-C NFs-2	−15.0	6.0	−11.5	0.8

**Table 2:** Typical Fe-based materials for EM wave absorption reported in related literatures.

Samples	Mass Ratio (wt%)	Optimal *RL* Values (dB)	Matching Thickness (mm)	Frequency Range *RL* ≤ −10 dB (GHz)	Ref
Fe_3_C/HCNTs	50	−21.5	2.0	4.0	^12^
C/Fe_3_O_4_ nanorods	55	−27.9	2.0	5.8	^17^
Fe–C nanofibers	50	−44.0	3.0	1.7	^24^
Fe-Fe_3_C/C microspheres	25	−18.8	1.5	4.0	^56^
Fe_3_C/graphitic carbon	70	−26.0	2.0	4.0	^57^
Fe_3_C/C nanocapsules	40	−38.0	1.9	2.0	^58^
Laminated RGO/Fe_3_O_4_	30	−26.4	4.0	2.0	^60^
hollow Fe_3_O_4_−Fe/G	18	−30.5	2.0	6.2	^62^
Solid Fe_3_C/N-C NFs-2	10	−34.9	6.0	6.2	This work
Macroporous Fe_3_C/N-C NFs	10	−22.1	2.0	4.8	This work

## Conclusions

In summary, solid and macroporous Fe_3_C/N-doped carbon nanocomposites have been successfully obtained by coupling electrospinning with heat treatment in an argon atmosphere. The nanocomposites possess superior microwave absorption capability at a lower doping content (10 wt%). The solid Fe_3_C/N-C NFs display the optimal *RL* value of −33.4 dB at 3 mm. The lower *RL* value can be attribute to the appropriate impedance matching, which can be achieved by changing the formation of the absorber and Fe_3_C content. The solid Fe_3_C/N-C NFs-2 present an optimal *RL*_min_ of −34.9 dB with the widest EAB of 6.2 GHz. The minimum *RL* value of the macroporous Fe_3_C/N-C NFs-1 is up to −32.8 dB at 4.2 GHz. The *RL* value of the macroporous Fe_3_C/N-C NFs exceeds −10 dB in the 3.6–18.0 GHz for the thickness of absorber layer between 2 and 6 mm. These Fe_3_C/N-doped carbon hybrid nanofibers are promising full-band EM-wave absorbers with low density and less doped metal.

## Methods

All chemicals were analytical pure and used without any pre-purification. Polyacrylonitrile (PAN, Mw = 150,000) and Iron acetylacetonate (97%) were purchased from Sigma-Aldrich Company. N, N-dimethyl formamide (DMF, 99.5%), Ethyl orthosilicate (TEOS), ammonium hydroxide (NH_3_·H_2_O, 25%), ethanol (C_2_H_5_OH, 99.7%) and sodium hydroxide (NaOH, 96%) were obtained from Beijing Chemical Reagent Co. Ltd.

### Preparation of solid Fe_3_C/N-doped carbon nanofibers

Typically, 1.0 g PAN and 0.8 g iron acetylacetonate were dissolved in 10 mL DMF, followed by slowly mixing under vigorously stirring for 24 hours. Next, the precursor solution was put in a syringe for spinning. The applied positive high voltage was maintained at 12 kV and the negative high voltage was maintained at −4 kV. The receiving distance was 15 cm and the feeding rate was controlled at 0.1 mL/min. The as-spun fibers were calcined at 280 °C for 3 h and then heated up to 800 °C with a heating rate of 1 °C/min in an Ar atmosphere. The sample obtained was recorded as solid Fe_3_C/N-C NFs. Besides, the solid Fe_3_C/N-doped carbon nanofibers with the different amount of iron acetylacetonate (0.6 and 1.0 mg, respectively) were prepared and named as solid Fe_3_C/N-C NFs-1 and solid Fe_3_C/N-C NFs-2, respectively.

### Preparation of macroporous Fe_3_C/N-doped carbon nanofibers

The template (silica microspheres) was synthesized according to the description by Stöber *et al*.^11^. The macroporous Fe_3_C/N-doped carbon nanocomposites were prepared using the similar synthetic process with 1.2 g previously synthesized silica template in the spinning dope. The obtained precursor fibers were placed in sodium hydroxide solution and stirred for 2 h to remove the template, followed by washing with deionized water and desiccation. The sample gained was recorded as macroporous Fe_3_C/N-C NFs. Besides, the macroporous Fe_3_C/N-doped carbon nanofibers with the different amount of iron acetylacetonate (0.6 and 1.0 mg, respectively) were prepared and named as macroporous Fe_3_C/N-C NFs-1 and macroporous Fe_3_C/N-C NFs-2, respectively.

### Preparation of pure N-doped carbon nanofibers

The pure N-C NFs were obtained by the similar process as solid Fe_3_C/N-doped carbon nanofibers synthesization without adding metal salt precursors.

### Materials characterization

The three solid Fe_3_C/N-doped carbon nanofibers and three macroporous Fe_3_C/ N-doped carbon nanofibers have almost the same morphology, structure and magnetic performance, therefore the solid Fe_3_C/N-C NFs and macroporous Fe_3_C/N-C NFs are selected as the representative in each group to describe its morphology, microstructure and magnetic performance in details. The structure of the products was carried out by X-ray diffraction (XRD). Field-emission scanning electron microscopy (FESEM, Hitachi SU-8010), high-resolution transmission electron microscopy (HRTEM, JEM-2010), scanning transmission electron microscope (STEM), energy-dispersive X-ray spectroscopy (EDX) and energy-dispersive spectrometer (EDS) analysis were used to characterize the morphology, microstructures and element content of the representative solid and macroporous nanofibers. X-ray photoelectron spectroscopy (XPS) spectra were characterized by ESCALAB 250Xi spectrometer (Thermo Fisher). Raman spectra were measured by a Raman spectrometer. The hysteresis loops were achieved by superconducting quantum interference device (SQUID-MPMS3) at room temperature.

### Measurement of electromagnetic parameters and absorbing performance

The value of complex permittivity (*ε*′, *ε″*) and permeability (*μ*′, *μ″*) of the as-synthesized seven products were evaluated via a network analyzer (Model No. HP-E8362B, Agilent, frequency range 1.0 GHz to 18.0 GHz). The measurement samples were prepared by homogeneously milling the powders (10 wt%) in paraffin and then pressing them into a ring of 7.0 mm external diameter, 3.04 mm inner diameter, 2.0 mm thickness^59,60^. The EM-wave absorbing ability of the materials were computed by equations (1) and (2)^61,62^.

1 $${Z}_{{in}}={Z}_{0}\sqrt{{\mu }_{r}/{\varepsilon }_{r}}\,{\tan }h[j({2}{\pi }{fd}/{c})\sqrt{{{\mu }}_{r}{\varepsilon }_{r}}]$$

2 $${RL}({dB})={20log}|({Z}_{{in}}-{Z}_{{0}})/({Z}_{{in}}+{Z}_{{0}})|$$

where d, *f*, c, Zin and Z_0_ represent the absorber thickness, microwave frequency, light velocity, input characteristic impedance and air impedance, respectively

## Electronic supplementary material

Supporting information
