# Supplementary material for: Solid and macroporous Fe3C/N-C nanofibers with enhanced electromagnetic wave absorbability
Source: Sci Rep. 2018 Nov 15;8:16832. doi: 10.1038/s41598-018-35078-z (PMC6237827; doi:10.1038/s41598-018-35078-z)
Supplement: Supplementary file 1 — Supporting information [file 41598_2018_35078_MOESM1_ESM.pdf]

Supporting information for
Solid and macroporous Fe_3C/N-C nanofibers with enhanced electromagnetic wave absorbability

Huihui Liu,[1] *Yajing Li,*[1] *Mengwei Yuan,*[1] *Genban Sun,*[*, 1, 2] *Qingliang Liao,*[*, 2] *Yue Zhang*[*, 2]

[1] Beijing Key Laboratory of Energy Conversion and Storage Materials and College of Chemistry, Beijing Normal University, Beijing 100875, China.

[2] State Key Laboratory for Advanced Metals and Materials, School of Materials Science and Engineering, University of Science and Technology Beijing, Beijing, 100083, China.

Correspondence and requests for materials should be addressed to G.S. (email: gbsun@bnu.edu.cn), Q.L. (email: liao@ustb.edu.cn) or Y.Z. (email: yuezhang@ustb.edu.cn).

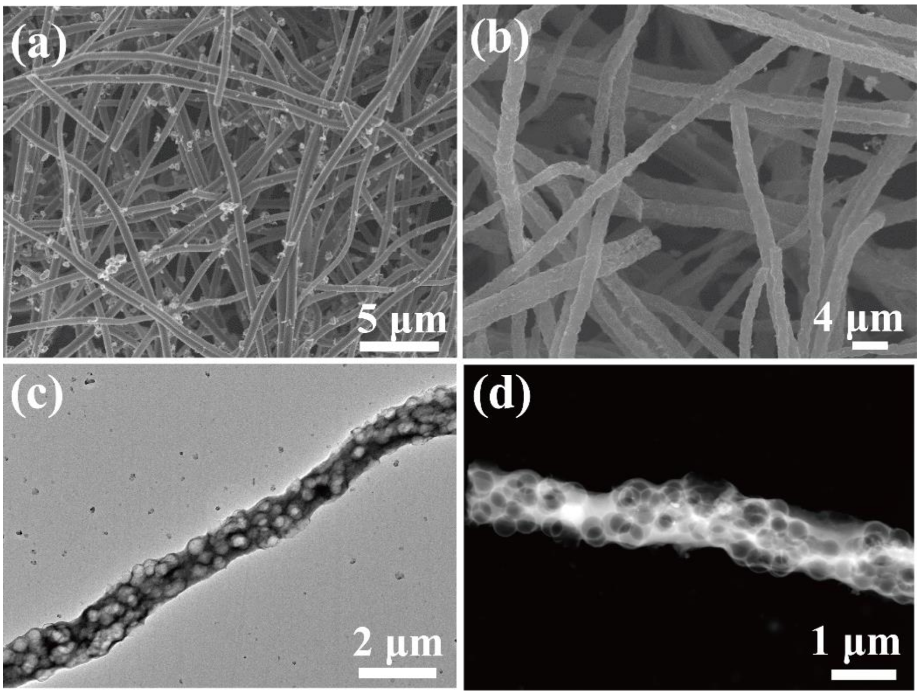

Figure S1. SEM images of (a) solid Fe_3C/N-C NFs, (b) macroporous Fe_3C/N-C NFs, (c) TEM images of macroporous Fe_3C/N-C NFs, (d) STEM images of macroporous Fe_3C/N-C NFs.

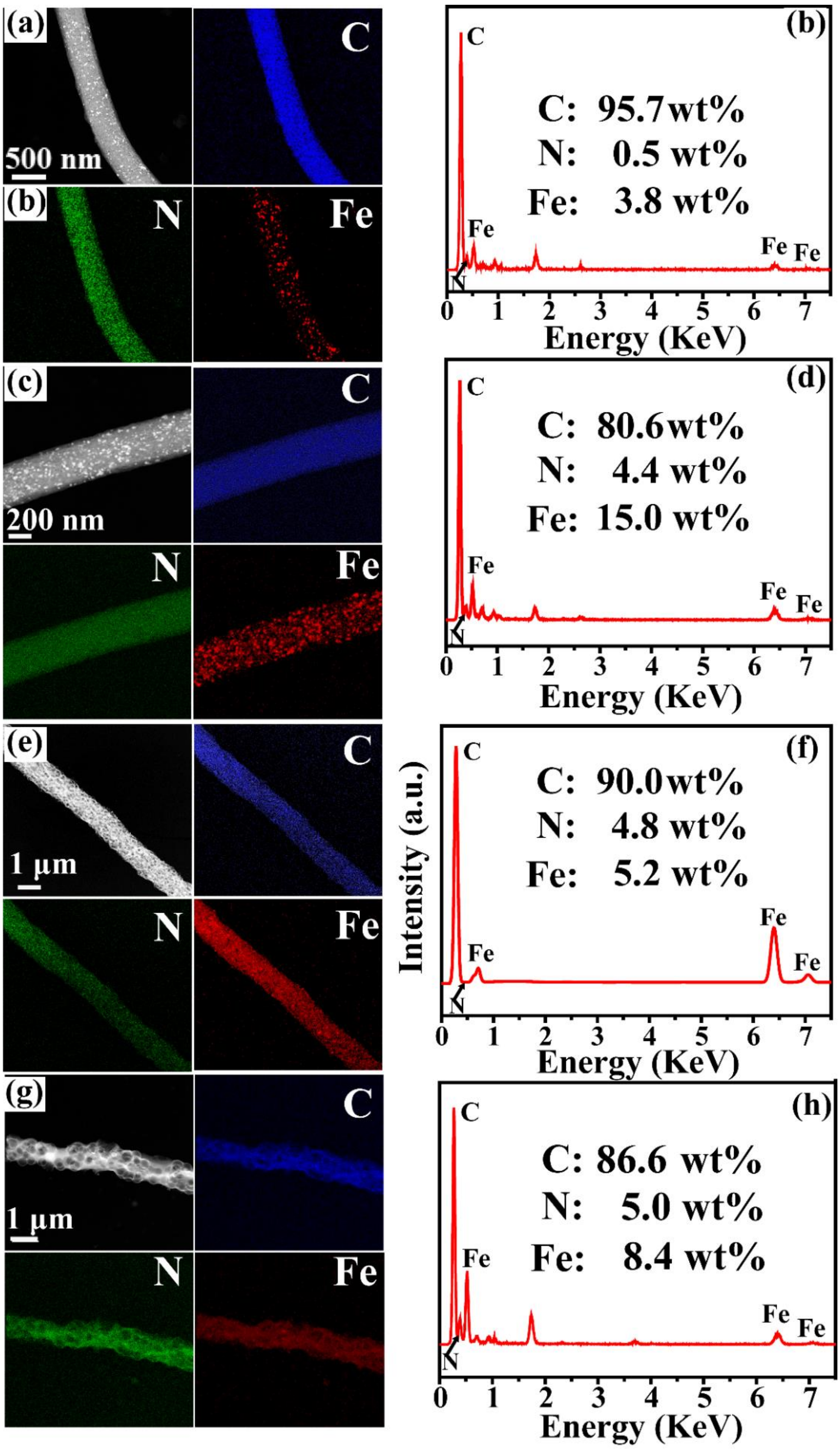

Figure S2. (a) (c) (e) & (g) EDX mapping images, (b) (d) (f) & (h) EDS spectra of solid Fe_3C/N-C NFs-1, solid Fe_3C/N-C NFs-2, macroporous Fe_3C/N-C NFs-1, macroporous Fe_3C/N-C NFs-2.

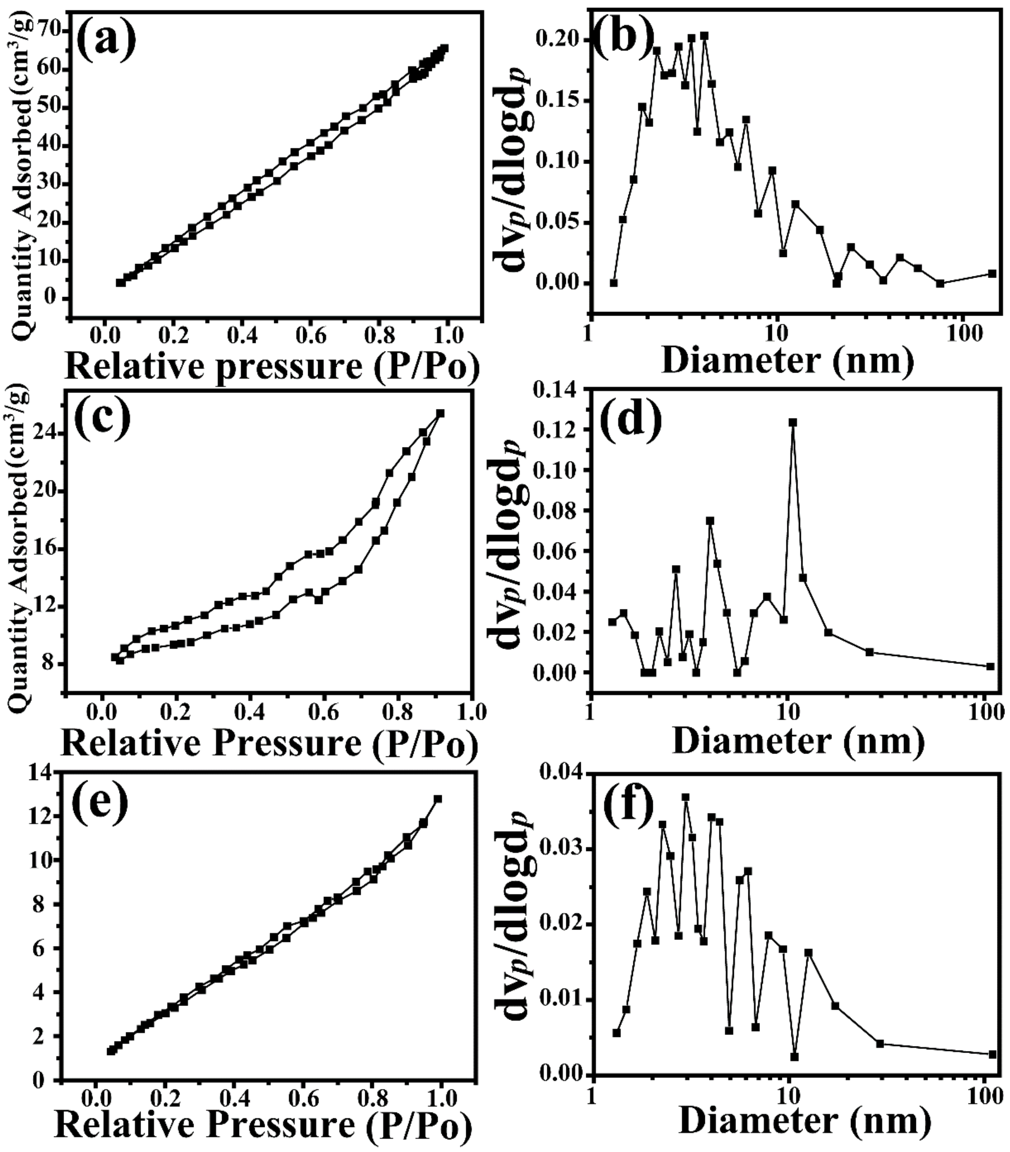

Figure S3. (a) & (c) & (e) nitrogen adsorption/desorption isotherms, (b) & (d) & (f) the pore size distributions of solid and macroporous Fe_3C/N-C NFs, pure N-C NFs, respectively.

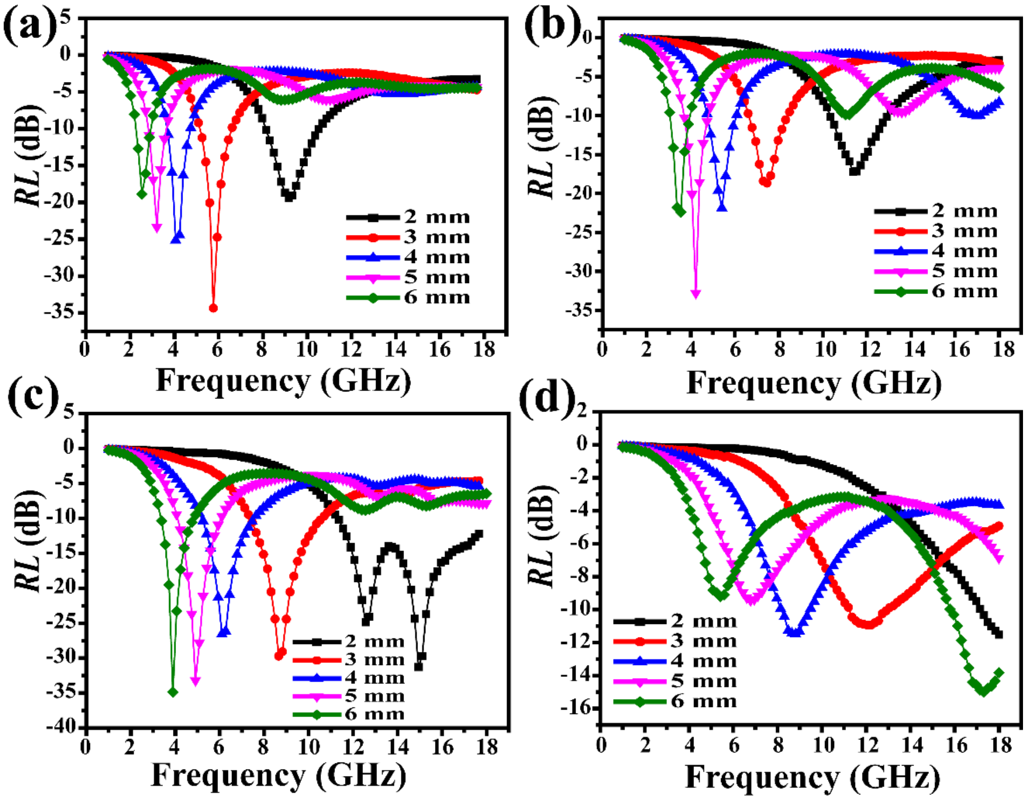

Figure S4. Reflection loss in the frequency range of 1−18 GHz: (a) solid Fe_3C/N-C NFs-1 (b) macroporous Fe_3C/N-C NFs-1 (c) solid Fe_3C/N-C NFs-2 (b) macroporous Fe_3C/N-C NFs-2 with 10 wt % filler content.
